# Editorial: Neuroinflammatory response and brain-peripheral crosstalk after stroke

**DOI:** 10.3389/fimmu.2022.1096185

**Published:** 2022-11-25

**Authors:** Devin W. McBride, Lei Huang, Qing-Wu Yang, Yujie Chen

**Affiliations:** ^1^ The Vivian L. Smith Department of Neurosurgery, The University of Texas Health Science Center at Houston, Houston, TX, United States; ^2^ Department of Neurosurgery, Division of Physiology, Loma Linda University, Loma Linda, CA, United States; ^3^ Department of Basic Sciences, Division of Physiology, Loma Linda University, Loma Linda, CA, United States; ^4^ Department of Neurology, Xinqiao Hospital, Third Military Medical University (Army Medical University), Chongqing, China; ^5^ Department of Neurosurgery, Southwest Hospital, Third Military Medical University (Army Medical University), Chongqing, China

**Keywords:** stroke, ischemia, hemorrhage, neuroinflammation, brain-peripheral crosstalk

Stroke, a devastating neurological disorder, is a leading cause of death and disability worldwide. Regardless of stroke subtype, following the initial insult, numerous inflammatory mechanisms are initiated by the resident cells (*i.e.* microglia, astrocytes, and pericytes) to propagate the inflammatory cascade and recruit peripheral immune cells to the site of injury.

It is well-understood that inflammation has two faces: pro-inflammatory and anti-inflammatory. Following stroke, pro-inflammatory cascades lead to worse outcome, whereas the anti-inflammatory cascades are focused on repair and regeneration. Thus, a goal has been to develop therapeutic targets which attenuate the deleterious pro-inflammatory pathways and bolster anti-inflammatory mechanisms. Of growing interest is the regulation of the crosstalk between the brain and peripheral immune systems as a way to improve outcomes after stroke. The goal of this Research Topic is to provide a collection of papers which focus on understanding neuroinflammation and the brain-peripheral immune crosstalk after stroke. In particular, the following sections were highlighted in this Research Topic.

## The role of innate immunity in the pathogenesis of post-stroke neuroinflammation

Microglia are the innate immune cells that are resident to the brain. Similar to their systemic counterparts (macrophages), microglia are in a resting state, and upon cytokine stimulation, can become either pro-inflammatory M1 or anti-inflammatory M2 phenotypes. Two manuscripts in this collection present mechanisms involved in changing microglia from the pro-inflammatory M1 phenotype to the anti-inflammatory M2 phenotype. Using experimental models of subarachnoid hemorrhage, these manuscripts suggest that multiple mechanisms can regulate microglia phenotype switching. More specifically, agonism of either the RARα receptor (Tian et al.) or SIRT1 (Xia et al.) can promote microglia to polarize towards the M2 phenotype, leading to less early brain injury and pro-inflammation, and ultimately, better outcomes.

## Different functions of neuroinflammation in the acute and chronic phases of stroke

It is well-understood that acute systemic inflammation after an insult is primarily pro-inflammatory. However, in the following days to weeks, as the pro-inflammatory response subsides, an anti-inflammatory environment increases to promote healing and repair; neuroinflammation is no different. In this collection, there are five manuscripts investigating several functions of neuroinflammation after stroke. In a model of bilateral common carotid artery ligation, Gao et al. reports on the changes to microglia morphology, as well as changes to gene transcription, for various lengths of occlusion. Of note, occlusion for 30 minutes caused microglia to lose their ramifications but did not affect overall microglia density, as opposed to 60 minutes of occlusion which, in addition to de-ramification, caused decreased microglia density and caused microglia to be pro-proliferative. Furthermore, 60 minutes of occlusion caused upregulation of pathways involved in apoptosis, cell mitosis, and inflammation.

TLR4 has been highlighted as a major receptor involved in the activation of numerous pro-inflammatory cascades after stroke, and two papers in this collection study the impact of TLR4 on stroke outcomes. Specifically, platelet-TLR4 was found to be a key protein involved in the formation of neutrophil extracellular traps (large web-like strands of cleaved DNA and proteins released by bursting neutrophils) (Peña-Martínez et al.). Additionally, neutrophil-TLR4 was observed to be crucial for neutrophil aging and circadian fluctuation. Interestingly, knockdown of TLR4 from myeloid cells reduced infarction after stroke as a result of less reactive oxygen species (Durán-Laforet et al.).

Yuan et al., using a chronic cerebral hypoperfusion model, investigated the role of astrocyte adenosine A2a receptor as a mechanism to inhibit pro-inflammation. Of note, the data suggests that the STAT3/YKL-40 pathway is downstream of the adenosine A2a receptor in astrocytes and mediates inflammatory response. In another paper, Zheng et al. reported on the pro-inflammatory role of neuronal AIM2 inflammasome, and observed that inhibition of Pannexin-1 reduced AIM2 inflammasome, thereby improving outcome after experimental subarachnoid hemorrhage.

## The influence of peripheral factors such as microbiota and cytokines on neuroinflammation

The role of peripheral factors in contributing to neuroinflammation is crucial to understand as modulating only the central inflammation is just part of the battle. Two papers included in this collection investigate peripheral factors. Amantea et al. investigated the role of ischemic preconditioning in attenuating the pro-inflammatory response after ischemic stroke. Specifically, the study observed that occlusion of the middle cerebral artery for 15 minutes was capable of priming the spleen and myeloid cells for a severe middle cerebral artery occlusion which occurred three days later. Of note, myeloid cells were observed to be polarized towards the anti-inflammatory M2/N2 phenotype by ischemic preconditioning which resulted in reduced infarction and improved outcomes after stroke in mice. The study by Wu et al. reported that inhibition of the triggering receptor expressed on myeloid cells 1 (TREM1) reduced neuroinflammation and improved outcomes after subarachnoid hemorrhage. Their data indicates that TREM1 inhibition promotes the anti-inflammatory phenotype of microglia, as well as decreasing the formation of neutrophil extracellular traps.

## Mechanisms underlying the dysfunctions of peripheral organs and the post-stroke brain-peripheral crosstalk involving brain-spleen axis, neurogenic lung diseases, cerebro-cardiac syndrome

The mechanisms causing injury to peripheral organs after stroke is understudied. Here, a collection of four articles contribute to this topic. Although neural stem cell therapy has been studied as a treatment for stroke, the impact of transgenic neural stem cells on organs and the inflammatory system remains unknown. Wei et al. investigate if transgene-modified neural stem cells can cause a reduced immune response and less damage to peripheral organs in a rat model. Interestingly, the modified neural stem cells did not induce a significant immune response compared to the non-modified neural stem cells.

Kim et al. examined the role of the hypothalamic-pituitary-adrenal axis in stroke outcome of diabetic mice. Diabetic patients have a dysregulated hypothalamic-pituitary-adrenal axis, and since diabetes is a risk factor for stroke, the hypothalamic-pituitary-adrenal axis may play a role in stroke outcome. In brief, their findings suggest that inhibition of glucocorticoid synthesis after stroke can attenuate ischemic brain injury and improve outcome in diabetic mice.

In large-urban cities, air pollution can be a major problem which may lead to lung dysfunction and inflammation. In a unique model of minor lung injury by mimicking air pollution, Huuskonen et al. investigated the impact of inhaled particulate matter on outcome of mice subjected to cerebral hypoperfusion. The authors observed that while nanoparticulate matter inhalation alone did not alter cerebral blood flow or blood-brain barrier integrity, it exacerbated the effects of chronic cerebral hypoperfusion on white matter injury and blood-brain-barrier permeability in comparison to unpolluted air inhalation. Furthermore, the nanoparticulate matter caused white matter demyelination irrespective of cerebral injury.

Jing et al. reported that lncRNA GAS5 was higher in patients experiencing cardiac arrest and cardiopulmonary resuscitation. Similarly, brain lncGAS5 was increased in mice subjected to the cardiac arrest and resuscitation. As lncRNA GAS5 reduces miR-137, the authors observed that either lncRNA GAS5 silencing or mir-137 over-expression attenuated neuroinflammation and promoted the anti-inflammatory M2 microglia phenotype in mice post cardiac arrest and resuscitation.

## Novel diagnosis or management strategies of post-stroke neuroinflammation

Diagnosing and managing inflammatory response after stroke is a major goal. As such, several articles in this collection have identified targets as either biomarkers or treatment of post-stroke inflammation.

The clinical study by Liu et al. investigated the possible correlation between outcome from acute basilar artery occlusion and a major complication: malignant cerebellar edema. In their study of 329 stroke patients, the authors report that malignant cerebellar edema was associated with worse outcome. Interestingly, a high neutrophil count at admission correlated with higher incidence of malignant cerebellar edema and thus poorer outcome. The work by Yang et al. in ischemic stroke patients examined eosinophil counts before and after intravenous thrombolysis and observed that a drastic decrease in eosinophil count of greater than 75% was associated with a 2.5 times higher risk for poor outcome and more than a 13-fold increased risk of death. Their work suggests that eosinophil counts may improve the predictive ability of outcome when combined with other predictive algorithms. Guo et al. observed that ischemic stroke patients who had low levels of Axl were more likely to develop hemorrhagic transformation, and, using an experimental model, they found that Axl may be a treatment for stroke. In their experimental model, rats administered Axl had less hemorrhagic transformation and reduced blood-brain barrier disruption. Henry et al. measured plasma short chain fatty acids in ischemic stroke patients and found that, although short chain fatty acids did not correlate with admission status, infarction, or edema volume, select short chain fatty acids were negatively correlated with recovery and positively correlated with pro-inflammation.

Regarding management and treatment of post-stroke inflammation, Xu et al. examined if inhibiting CD47 could improve blood clearance following subarachnoid hemorrhage. Indeed, mice subjected to subarachnoid hemorrhage had quicker clearance of subarachnoid blood following administration of a CD47 antibody, resulting in less neuroinflammation. Another article observed that a flavonoid could reduce microglia activation and neutrophil infiltration after intracerebral hemorrhage in mice, thereby reducing neuroinflammation and blood-brain barrier disruption, subsequently improving behavioral outcomes (Gu et al.). Finally, an article by Li et al. suggests that co-administering a sphingosine 1-phosphate receptor antagonist with tPA can improve thrombolysis.

In addition to the original research published in this Research Topic, several reviews are published which discuss the response to stroke by the brain-peripheral axis (Bourhy et al.; Shaheryar et al.; Yu et al.) by T-Cells (Zhang et al.), and by neutrophils (Yusuf et al.; Wu et al.), as well as the roles of oxidative stress (Zhang et al.; Zhu et al.), thromboinflammation (Sun and Langer), and post-stroke angiogenesis (Ma et al.)

In the future, understanding the precise molecular interactions of neuroinflammation and the interactions between the brain and peripheral immune system may yield therapeutic targets to mitigate the detrimental effects of inflammation on post-stroke outcomes.

## Author contributions

All authors listed have made a substantial, direct, and intellectual contribution to the work and approved it for publication.

## Funding

This work was supported by State Key Laboratory of Trauma, Burn and Combined Injury (SKLYQ202002 to YC), the Brain Aneurysm Foundation (DWM), and the National Institutes of Health (5R01NS115887 to DWM).

## Conflict of interest

The authors declare that the research was conducted in the absence of any commercial or financial relationships that could be construed as a potential conflict of interest.

## Publisher’s note

All claims expressed in this article are solely those of the authors and do not necessarily represent those of their affiliated organizations, or those of the publisher, the editors and the reviewers. Any product that may be evaluated in this article, or claim that may be made by its manufacturer, is not guaranteed or endorsed by the publisher.

